# Use of Public Automated External Defibrillators in Out-of-Hospital Cardiac Arrest in Poland

**DOI:** 10.3390/medicina57030298

**Published:** 2021-03-22

**Authors:** Przemysław Żuratyński, Daniel Ślęzak, Sebastian Dąbrowski, Kamil Krzyżanowski, Wioletta Mędrzycka-Dąbrowska, Przemysław Rutkowski

**Affiliations:** 1 Department of Medical Rescue, Faculty of Health Sciences, Medical University of Gdańsk, Dębinki 7, 80-211 Gdańsk, Poland; przemyslaw.zuratynski@gumed.edu.pl (P.Ż.); sebastian.dabrowski@gumed.edu.pl (S.D.); kamil.krzyzanowski@gumed.edu.pl (K.K.); 2 Department of Anaesthesiology and Intensive Care Nursing, Medical University of Gdańsk, 80-210 Gdańsk, Poland; wioletta.medrzycka-dabrowska@gumed.edu.pl; 3 Department of Internal and Pediatric Nursing, Faculty of Health Sciences, Medical University of Gdańsk, 80-211 Gdańsk, Poland; przemyslaw.rutkowski@gumed.edu.pl

**Keywords:** automated external defibrillators, out-of-hospital cardiac arrest, public access to defibrillation

## Abstract

*Background and objectives:* National medical records indicate that approximately 350,000–700,000 people die each year from sudden cardiac arrest. The guidelines of the European Resuscitation Council (ERC) and the International Liaison Committee on Resuscitation (ILCOR) indicate that in addition to resuscitation, it is important—in the case of so-called defibrillation rhythms—to perform defibrillation as quickly as possible. The aim of this study was to assess the use of public automated external defibrillators in out of hospital cardiac arrest in Poland between 2008 and 2018. *Materials and Methods:* One hundred and twenty cases of use of an automated external defibrillator placed in a public space between 2008 and 2018 were analyzed. The study material consisted of data on cases of use of an automated external defibrillator in adults (over 18 years of age). Only cases of automated external defibrillators (AED) use in a public place other than a medical facility were analysed, additionally excluding emergency services, i.e., the State Fire Service and the Volunteer Fire Service, which have an AED as part of their emergency equipment. The survey questionnaire was sent electronically to 1165 sites with AEDs and AED manufacturers. A total of 298 relevant feedback responses were received. *Results:* The analysis yielded data on 120 cases of AED use in a public place. *Conclusions:* Since 2016, there has been a noticeable increase in the frequency of use of AEDs located in public spaces. This is most likely related to the spread of public access to defibrillation and increased public awareness.

## 1. Introduction

According to Olson et al., sudden cardiac arrest (SCA) accounts for more than half of all cardiovascular deaths and is the first sign of heart disease in 50% of these individuals [1]. Cardiac arrest, according to Chugh et al., should be treated as a common and massive public health problem worldwide [2]. SCA can occur in any location. Out-of-hospital cardiac arrest (OHCA) according to Engdahl et al. most commonly occurs at home, which is confirmed by Alqahtani et al. adding the street and public places and the workplace [3,4]. The frequency of OHCA episodes varies widely around the world (higher in North American countries than in Europe, Asia, or Australia). The aggregate value varies between 24–186 per 100,000 persons/year. Sudden cardiac arrest is more common among the male population. Analyzing the age of the victims, the highest number of OHCA episodes is observed in older people, i.e., over 50 years of age. It is relatively rare in people aged 18–40 years [5,6,7]. Mortality from SCA is high worldwide. National medical records indicate that approximately 350,000–700,000 people die annually from SCA. More than half of these deaths occur in the prehospital setting [6]. SCA requires immediate resuscitation, i.e., a combination of chest compressions and assisted ventilation. The guidelines of the European Resuscitation Council (ERC) and the International Liaison Committee on Resuscitation (ILCOR) indicate that in addition to resuscitation, it is important—in the case of the so-called defibrillation rhythm—to perform defibrillation as soon as possible. Depolarization of all myocardial cells at the same time interrupts the “reentry” phenomenon, which is responsible for the development of arrhythmia and allows the sinus node to impose its rhythm on the whole heart [5]. Defibrillation can be performed using manual defibrillators, operated by the medical services, or automated external defibrillators (AEDs), whose simplicity of operation allows them to be used also by medically untrained people [4,8].

### Aim

The aim of this study was to assess the use of public automated external defibrillators in out-of-hospital cardiac arrest between 2008 and 2018 in Poland.

## 2. Materials and Methods

### 2.1. Design

A retrospective analysis of 120 uses of an automated external defibrillator placed in public spaces between 2008 and 2018.

### 2.2. Participants and Procedure

The material studied included data on the use of automated external defibrillator in adults (over 18 years of age). The analysis included only cases of AED use in a public place, other than a healthcare institution, additionally excluding emergency services, i.e., the State Fire Service and Volunteer Fire Service, which have an AED as part of their emergency equipment. The study was based on a diagnostic survey and retrospective analysis of medical records. The survey questionnaire was sent to sites with an AED (Table 1). The addresses of the sites were generated on the basis of publicly available AED registers in Poland. Additionally, the survey questionnaire was sent to manufacturers and distributors of AEDs in Poland (Table 2).

The data obtained from the questionnaire survey, constituting specific cases of AED use, were detailed by retrospective analysis of selected medical records (data obtained from the reports of the emergency notification center (medical dispatch center), cards of medical rescue activities). The data were anonymized. Only the date and time of the event, age, and gender were taken into account.

### 2.3. Statistical Analysis

All material for the study was collected in Microsoft Office Excel spreadsheet for Windows systems. In order to answer the research questions posed, statistical analyses were conducted using R 3.5.3 software (R version 3.5.3 (Great Truth) of 2019). Using it, analysis of basic descriptive statistics was performed. Poissone testing of the data was performed, as the data collected were a specific number of events in a given specified time interval. According to the mathematical definition, data in the form of a number of events in a specific time interval (i.e., number of AED interventions/year) have a Poisson distribution. The classical threshold of α = 0.05 was considered as the significance level.

## 3. Results

### 3.1. Locations of AEDs Obtained from Publicly Available Registers and Maps

The authors took into account the period of deployment and use of the AED in 2008–2018, because the largest number of AED devices was purchased and deployed in 2005 as a result of the action (The Great Orchestra of Christmas Charity), but the lack of registration of AED use in Poland and the lack of 100% return allowed us to collect data only from 2010. During the project 1165 places where AEDs were installed were located. This number refers only to specific places and not to the total number of AEDs in Poland, as many institutions have more than 1 AED. When presenting the profile of a place where an AED is located it may be noticed that most addresses (198) correspond with commercial establishments (shopping centers, large-format shops). An equally frequent distribution of AEDs has been noted in cultural and entertainment institutions such as cinemas, theatres, opera houses, museums, art galleries, and libraries (176). The smallest number of locations was noted in places of worship i.e., church, monastery, place of religious congregation (13).

### 3.2. Analysis of the Survey Questionnaire Responses

The survey questionnaire was sent electronically to 1165 to sites with an AED device. A total of 326 (27.98%) feedback responses were received. 

Of the 326 replies sent, 311 (95.4%) were responses to the questions asked. 14 (4.6%) refused to answer the questions. 298 (95.82%) units responded that there was at least 1 AED device in their area. The remaining 13 (4.18%) sites did not have an AED device or were considered unfit for use. 54% of sites allow 24-h access to AEDs. The remaining AEDs are only available during unit operating hours. The devices are placed, for example, at customer/patient service points, security rooms. Only 20% of the 24/7 locations are located outside the building (e.g., building façade). The questionnaire was also sent by e-mail to 17 manufacturers and distributors of AEDs in Poland. The total number of returned answers-14 (82.35%). Nine entities refused to provide any information, citing commercial law and data protection in the broad sense.

Based on responses from a survey questionnaire sent to AED locations and AED manufacturers and distributors in Poland, data on 120 cases of AED use in a public place between 2008 and 2018 was obtained.

### 3.3. Analysis of AEDuse Interventions by Year, Month, Day of the Week, Season, and Time of Day

The number of AED interventions in particular years, months, days of the week, seasons and times of the day was analyzed (Table 3). In addition, on the basis of the collected data, the victims were characterized (age, gender), the place of AED use, and the rescuer.

Analyzing the amount of AED use in individual years, it can be seen that this number fluctuated between 2010 and 2016, but within a constant level. The number of interventions/year did not differ significantly between the years mentioned. The first significant increase was observed in 2017 (*p* = 0.006). When considering the number of AED uses per season, no significant differences were observed between them (*p* = 0.332). This means that the frequency of interventions was not significantly different between the seasons. When analyzing the use of AEDs in individual months, it can be seen that the number of interventions was highest in April and lowest in November. The difference between these months is statistically significant (*p* = 0.027). The highest number of interventions with AEDs occurred on Friday and the lowest on Sunday. The difference between the mentioned days of the week is statistically significant (*p* = 0.049). Considering the use of AED in individual times of the day, it can be observed that it is significantly lower between 8 p.m. and 8 a.m. In this time interval the number of interventions did not differ significantly between individual times of the day. In the hours 8:00–12:00, 12:00–16:00, and 16:00–20:00, the number of interventions was significantly higher than at the above-mentioned times (respectively: *p* = 0.013; *p* < 0.001; *p* = 0.043). It is also worth noting that the number of interventions between 12:00 and 16:00 was significantly higher than from 8:00 to 12:00 and between 16:00 and 20:00 (*p* = 0.015).

### 3.4. Characteristics of the Victim

The most frequent AED use was in the age group 50–60 years (31%), M = 57.27 years. Most AED interventions involved males (87.5%). The dominant group were travelers by various means of transport (30%). Equally high values were recorded among people classified as “customer/petitioner” of a commercial, banking or office establishment (23.33%) and “employee” (22.5%).

### 3.5. Characteristics of Where the AED Is Used

The most frequent use of AEDs was observed on public transport infrastructure (24.17%). A high rate was also recorded at factories and warehouses (workplace—17.5%) (Table 4).

### 3.6. Characteristics of the Person Who Used the AED

Those who used an AED while performing CPR were outnumbered by employees (n = 96) (i.e., security guard, police officer, medical staff, hotel staff, tram driver, pool attendant) over casual witnesses (n = 24).

### 3.7. Analysis of the Administration of Resuscitation Using an AED

Of 120 cases of AED use in OHCA, 76 required defibrillation (63.33%). In 37 cases (30.83%) defibrillation was not performed. Detailed information was not obtained from 7 AED interventions.

On the basis of 33 thoroughly documented resuscitation actions with the use of AED we can observe that the average time that elapsed from SCA to the use of AED oscillated around 3 min and 43 s (M=222.73 s; SD = 121.98 s). The maximum time that elapsed from SCA to AED use was 10 min (max = 600 s).

For 24 interventions, the first OHCA mechanism analyzed was ventricular fibrillation (VF) which accounted for 72.73%. One case was ventricular tachycardia without pulse (VT). Non-defibrillatory rhythms were identified in a total of 8 events (AS-n = 5, 15.15%; PEA-n = 3; 9.09%).

## 4. Discussion

Prehospital cardiac arrest is a major medical and social problem and the leading cause of death in Europe and the United States. It requires the earliest possible resuscitation. Despite the increasing availability of AEDs, their use before the arrival of emergency services is only 2–5%, and the reasons for such infrequent use of AEDs are not entirely clear [9]. Three elements play a key role in reducing OHCA mortality: the defibrillator, the witness to the event, and the medical dispatcher. Only the timely action of the first person on the scene, the competence of the medical dispatcher and the availability of an AED can significantly affect the survival of a person with cardiac arrest [5]. The system of public access to defibrillation in Poland has been continuously developed since 2000. The collected material shows that between 2008 and 2018 there were 120 cases of use of an AED located in a public place in Poland. Given the low response rate from AED sites, it is reasonable to believe that the number of AED uses was greater than 120. The number of uses of automatic defibrillators in Poland is increasing every year. It can be presumed that this is due to the growing awareness of society in the field of resuscitation activities, as well as the increasing number of AEDs. However, there is no scientific reference as no research was conducted.

Analyzing the number of AED interventions by month, day of week, season, and time of day, it can be seen that the highest number of AED uses was in April, while the lowest was in November. AEDs were used more often on Fridays than on Sundays. The time of day may also influence the number of AED uses. It was noted that there were the highest number of OHCAs between 12 pm and 4 pm. Translating this into a day/night split, i.e., from 8:00–20:00/20:00–8:00, 70% of cases were during the day and 30% at night. In a study conducted by Bagai et al. from 2005 to 2010, significant variability was noted in the incidence of OHCA according to time of day (*p* < 0.001), day of week (*p* < 0.001), and month of year (*p* < 0.001), with the highest incidence occurring during the day from Friday to Monday in December [10]. Similar findings were obtained by Nadolny et al., noting that SCA occurred most frequently in the first quarter of the year, between 07:00 and 19:00 [11]. It can be concluded that low rates of AED use on Sundays and at night partly find their cause in the location of the device itself. A significant number of AEDs are located in buildings where no one is present at the weekend and at night. There is also a lack of potential patients who may suffer cardiac arrest, for example in the workplace. Hansen et al. noted that in Copenhagen, Denmark, most AEDs were available during the day on all days of the week. Only 9.1% (n = 50) of all AEDs were available 24 h a day, 7 days a week [12]. On the other hand, Lin Zhang et al. in their study indicated that 40% of AEDs mapped on three districts in Shanghai were in fact-for safety reasons-not available to the public, due to their placement in school buildings and government institutions [13]. Similar availability of AEDs was demonstrated by Agerskova et al. in the analysis of the OHCA in Copenhagen [14].

In order to achieve the expected results, i.e., to reduce OHCA mortality, the widest possible permanent access to public defibrillation should be sought [12]. As highlighted by Karlsson et al. in their paper, the 30-day survival of people with OHCA almost doubled when, during cardiac arrest, the nearest accessible AED was within 200 m of the scene [15].

The behavior of the first person on the scene is important in reducing OHCA mortality. It is up to them to initiate the resuscitation procedure. Apart from notifying the emergency services and starting resuscitation, it is important to provide and correctly use the AED. In the study group, it was most often the person employed (80%) in the place where the cardiac arrest occurred. Security staff dominated. Apart from them there were police officers, medical staff, hotel staff, tram driver, swimming pool attendants. These were usually people who were responsible for the AED at the moment and had training in its use. The willingness to use an AED by an OHCA witness, in some work, is very low and may be due to several factors. Brooks et al. in a survey study showed that only 2.1% of respondents between the ages of 9 and 90, would be prepared to use an AED in a cardiac arrest situation. The reasons for such low willingness to use AEDs were: lack of knowledge of how the device works, feeling of discomfort when using it, fear of possible harm to the patient or legal issues. [16]. Sudden cardiac arrest is more common among men. This is supported by many studies. The male gender is at risk for all heart diseases [1,2,17]. In the material presented, 87.5% of AED interventions concerned men. When analyzing the age group, it was observed that the highest rate of AED use occurred in the age group of 50–60 years (31%), which is included in the mean of 57.27 years and the median of 56.5 years.

The largest group in which OHCA with AED intervention occurred were travelers (30%). This is most probably the result of high availability of defibrillators in buildings of air, railway, bus or public transport infrastructure. This group includes cases of AED use on board trains, airports and railway stations. According to the guidelines of ERC or AHA, these are the places with the highest risk of OHCA. It should be an aim to have at least 1 automated defibrillator in these places. This was confirmed by Caffrey et al. O’Rourke et al. Page et al. and Weaver et al. who published studies on the use of AEDs, among others at airports and on board aircraft [18,19,20,21]. Another group consisted of customers/patients of retail, banking, or office establishments (23.33%) and employees (22.5%). These data can be contrasted with the profile of where the defibrillator was used. The majority of cases were on public transport infrastructure, followed by workplaces and public-service premises. This fact is supported by the ERC and AHA guidelines as places at high risk of OHCA [5,8]. The mean total device delivery time was 96 s with a maximum of 144 s. Telec et al. investigated the assessment of actual AED availability and the evaluation of possible sources of defibrillation delays. From a selected group of 200 sites, they chose 78 sites and sent volunteers who had no knowledge of the location of the AED at that site [22]. Based on the OHCA simulation, the volunteers had to respond appropriately including, but not limited to, using an AED. The devices were located within a range of 2–163 m from the incident site. In the material studied, the mean time of electrode application was 35.36 s. The longest recorded time reached 115 s. This prolonged time is puzzling, as sticking the electrodes takes a maximum of 10–15 s.

The use of AEDs in Poland and worldwide should be continuously addressed. Systemic programs concerning the development of public access to defibrillation should be pursued. As far as national decision makers are concerned, the key initiative should be the creation of a central register of AEDs and a system for reporting their use. It is worth being open to new technological solutions. For example, drones equipped with AEDs could improve patient survival and quality of life [23,24]. In their work, Claesson at al. showed that in non-urban areas, the delivery of AEDs by drones is safe and faster than the arrival of an emergency medical services team, with an average time saved of 19 min [25]. In some countries, e.g., France or the Netherlands, systems have been developed and locally implemented to notify volunteers via SMS about the place of cardiac arrest and the nearest AED [26,27]. It is also worth thinking about reporting the use of AEDs on medical emergency cards.

## 5. Limitations

As presented in the methodology of the study, in order to achieve the objective set, a questionnaire developed questions addressed to the entities where AEDs were located, as well as manufacturers, distributors of AEDs. The numbers presented were for responses given in writing and by telephone. Among the feedback, one could see responses that negatively related to providing information to factual questions, such as “Due to this and due to our Company’s procedures, we are unfortunately unable to complete and send the survey you submitted” or “Due to the fact that the AED device has not been used at our facility, we are unable to answer your question”. The units explained themselves with the protection of their good name, protection of personal data, and unwillingness to make public the so-called accidents at work. They did not accept the explanation that the fact of having an AED may put their institution in a good light, e.g., as an entity caring for the safety of the public or employees. Due to the lack of legislation on reporting the possession of AEDs they could not be forced to respond. 

## 6. Conclusions

The results obtained and conclusions drawn are based only on the research material collected, because not all the sites or manufacturers/distributors of AEDs in Poland answered the questions. Since 2016, there has been a noticeable increase in the frequency of use of AEDs located in public spaces. This is most likely related to the spread of public access to defibrillation and increased public awareness. There is no dependence of AED use on the season. There is a dependence in individual months (most in April, least in November), days of the week (most on Friday, least on Sunday), times of the day (most between 8:00 and 16:00, least between 20:00 and 04:00). The predominant place where AEDs are used is on public transport infrastructure and the casualty is the traveler. AED use in out-of-hospital settings is more common in male victims aged 50–60 years. Reporting of AED use in public places should be pursued.

## 7. Implications for Practice

In Poland, there is no legal basis for keeping a register of automated external defibrillators. There is a need to develop appropriate documents conditioning the process of reporting by owners the use of AEDs in out-of-hospital cardiac arrest conditions. The documentation constituting the basis for the intervention of the emergency medical services needs to be further developed—currently there is no record of resuscitation performed by a witness of the event or of the use of an AED.

## Figures and Tables

**Table 1:** Questions from a questionnaire targeting sites with an automated external defibrillator (AED) device.

L.p.	Content of the Question
1.	Is there an AED device(s) in the area you manage?
2.	Have any of the placed AEDs been used (2008–2018)?
3.	By whom was the AED used?
4.	Under what circumstances was the AED used? Please describe briefly.

**Table 2:** Questions from a questionnaire addressed to manufacturers and distributors of AEDs in Poland.

L.p.	Content of the Question
1.	Do you have knowledge of the use of an AED device (2008–2018)?
2.	Under what circumstances was the AED used? Please describe briefly.

**Table 3:** Frequency of AED interventions varied by year, month, day of week, season, and time of day.

		Number of AED Interventions/Year	95% CI		*p*-Value *
			LL	UL	
Frequency of AED interventions by year	2010	4	1.09	10.24	*p* = 0.006
	2011	2	0.24	7.22	
	2012	6	2.20	13.06	
	2013	3	0.62	8.77	
	2014	9	4.12	17.08	
	2015	8	3.45	15.76	
	2016	14	7.65	23.49	
	2017	34	23.55	47.51	
	2018	40	28.58	54.47	
Frequency of AED interventions by season	spring	11.6	7.77	16.66	*p* = 0.332
	summer	8.8	5.51	13.32	
	autumn	10.4	6.79	15.24	
	winter	12.0	8.10	17.13	
Frequency of AED interventions by month	January	13.2	6.59	23.62	*p* = 0.027
	February	12.0	5.75	22.07	
	March	9.6	4.14	18.92	
	April	19.2	10.97	31.18	
	May	8.4	3.38	17.31	
	June	8.4	3.38	17.31	
	July	7.2	2.64	15.67	
	August	8.4	3.38	17.31	
	September	14.4	7,44	25.15	
	October	9.6	4.14	18.92	
	November	6.0	1.95	14,00	
	December	12.0	5.75	22.07	
Frequency of AED interventions by day week	Monday	11.2	6.40	18.19	*p* = 0.049
	Tuesday	11.2	6.40	18.19	
	Wednesday	8.4	4.34	14.67	
	Thursday	11.2	6.40	18.19	
	Friday	15.4	9.65	23.32	
	Saturday	10.5	5.88	17.32	
	Sunday	7.0	3.36	12.87	
Frequency of AED interventions by time of day	24:00–4:00	1.2	0.15	4.33	*p* = 0.015
	4:00–8:00	4.8	2.07	9.46	
	8:00–12:00	14.4	9.23	21.43	
	12:00–16:00	23.4	16.64	31.99	
	16:00–20:00	12.6	7.80	19.26	
	20:00–24:00	5.4	2.47	10.25	

95% CI—confidence interval; LL and UL—lower and upper bounds of the confidence interval; * A *p*-value less than 0.05 (typically ≤ 0.05) is statistically significant. The classical threshold of α = 0.05 was considered as the significance level.

**Table 4:** AED device location profile (n = 120).

AED Usage Site Profile	*n*	%
Public transport infrastructure (railway/bus station, train station/bus stop, underground stations)	29	24.17
Place of work (factory, warehouse, mine, steelworks)	21	17.50
Airport/sea port	10	8.33
Shopping centre (estate shop, multi-store)	9	7.50
Nursing home	9	7.50
Public space, i.e., pavement, market, car park	8	6.67
Office/institution (e.g., Social Insurance Institution, Tax Office)	5	4.17
Sports centre (swimming pool, playground, stadium, sports hall, gym)	5	4.17
Detention/ Prison	4	3.33
Lakeside beach	4	3.33
State, governmental, territorial administration (ministry, town/municipality/county council)	4	3.33
Place of temporary accommodation (hotel, holiday home, hostel, hostel)	3	2.50
Bank branch	2	1.67
Cultural and entertainment institution (cinema, theatre, opera, museum, art gallery, library)	2	1.67
Urban/interurban transport (tram, bus, trolleybus, train, metro)-mobile	2	1.67
Church, monastery, place of religious assembly	2	1.67
Schools, universities	1	0.83

## Data Availability

Datasets used and analyzed during the current study are available from the corresponding author on reasonable request.
